# Machine learning models for chronic disease risks using complex survey data: the impact of sample weights

**DOI:** 10.3389/fdgth.2026.1834715

**Published:** 2026-08-10

**Authors:** Hyeonju Kim, Paul Rogers, Dong Wang

**Affiliations:** Division of Bioinformatics and Biostatistics, National Center for Toxicological Research, U.S. Food and Drug Administration, Jefferson, AR, United States

**Keywords:** chronic noncommunicable diseases, class imbalance, complex survey data, machine learning, NHANES, replicated weights methods, sample weights

## Abstract

Chronic noncommunicable diseases are the leading cause of deaths globally but can be preventable by early detection, screening, and treatment. Recent studies have used machine learning methods ignoring sample weights determined by a complex design framework, which leads to significant bias and ends up with the spurious conclusion in statistical inference. We have examined whether adjusting sample weights improves prediction and variable selection using two examples, hypertension and diabetes, from the National Health and Nutrition Examination Survey data. We used the traditional K-fold cross validation and its weighted counterparts, replicate weights methods, to finalize models fitted by four popular machine learning methods and two under-sampling approaches for handling class imbalance ad hoc. Due to lack of options managing complex sampling structures in existing machine learning software packages, We have also developed a r-MLSurvey that integrates the replicate weights methods into the machine learning methods as an extension of weighted L1-penalized logistic regression. The model performance was assessed by accuracy, sensitivity, specificity, the area under the receiver operating characteristic curve (AUC-ROC), and their counterparts for the weighted model evaluation. Results show that variable selection results differ by the under-sampling methods and sample weights’ adjustment; the weighted AUC, compared with the AUC, suggests final unweighted models either overestimate or underestimate the true population parameters. The sampling design also appears to provide moderate influence on accuracy for these relatively well-studied diseases, but the effect on selected variables’ significance varies by algorithm and class imbalance. Random forest would be preferred without users’ capability of adjusting sample weights. Careful selection of the final models is recommended for moderately class-imbalanced complex survey data to avoid biasedness. Our application to the machine learning algorithms offers the usefulness of complex survey data to achieve unbiased prediction in broader research disciplines in need of sufficiently large data.

## 1 Introduction

Noncommunicable Diseases (NCDs), also known as chronic diseases, are the foremost cause of death and disability worldwide, responsible for more than three out of four years with disability as well as 74% of all annual deaths, and require long duration or lifelong care. The main types of NCDs are cardiovascular diseases (CVD), cancers, chronic respiratory diseases, and diabetes, all of which constitute over 80% of all premature NCD deaths. One may note that hypertension itself is not only NCD but also a crucial risk factor for CVD and other chronic diseases. Socioeconomic factors, furthermore, by a country’s economic development, cultural factors, and social and health policies influence the survival of NCDs, which results in staggering economic costs with regard to lost productivity and soaring health-care costs. Managing NCDs includes detection, screening, and treatment of these diseases, with palliative care for people in need [1–5].

With the advance of modern computation technology and data science, the application of predictive models to medical domains using various machine learning algorithms to solve diagnostic and prognostic problems has become increasingly popular with some notable examples of success [6–11]. One critical challenge for building a useful predictive model is to locate suitable datasets of sufficient size. As a result, there has been a significant interest in using survey data in medical care and public health to build predictive models. An example of the health survey data is the National Health and Nutrition Examination Survey (NHANES) samples, collected by a complex, multi-stage, probability sampling design, widely used in epidemiological studies and health sciences research. The NHANES data has been used to study the prevalence of major diseases, their risk factors and is a part of national surveillance initiatives. This population-based design renders estimates from the data generalizable to the target population but needs the proper use of sample weights incorporated with design variables. It is known that ignoring survey sampling weights can cause significant bias, which leads to spurious conclusions in statistical inference. In particular, the NHANES program has yearly collected different population groups and health topics without interruption since early 1960s to accurately represent the health and nutritious status of everyone in the US [12–14]; therefore, it is useful for building predictive models for NCDs. Many publications have utilized health survey data and ML algorithms; most have simply built predictive models neglecting sample weights while the effect of sample weights on prediction performance has not been well studied. MacNell et al. (2023) also pointed out this inadequacy, which stemmed from the lack of ML software packages managing such adjustments, and placed an emphasis on implementing weighted algorithms. They additionally offered how to rescale model performance post-hoc with weighted outcome data based on gradient boosting (XGBoost) [15] to provide practical advantages in the absence of weighted algorithm configurations [13].

In this paper, we report an empirical investigation of effects of sample weights on the performance of final ML models using the NHANES data. Though our study is limited to two chronic diseases, results provide information on practical considerations on the impact of sampling weights when predictive models are developed using health survey data. We need to address that the traditional CV method is not suitable for complex survey data in model selection since a random split of dividing a dataset into K folds, followed by creating K training and test sets, does not reflect the sampling design. We instead employed “replicate weights” methods, which are typical approaches for survey data, to generate partially independent subsets of a sample by modifying the sample weights to replicate the original sample, so that the new subsamples correctly represent the finite population [16–18]. Iparragirre et al. proposed three new methods borrowing the idea of replicate weights methods: a design-based cross validation method (dCV), Split-sample Repeated Replication (split), and extrapolation (extrap). They applied these methods to LASSO–Least Absolute Shrinkage and Selection Operator, or $L_{1}$ penalization–regression for model selection, compared with other replicate methods as well as the unweighted simple random sample K-fold CV (unw-SRSCV), i.e., the traditional K-fold CV. They showed the need for adjusting sampling weights to modeling LASSO regression since the unw-SRSCV method selected unnecessarily complex models, eventually yielding poor performance; conversely, the dCV selected more parsimonious models under the assumption that the design effect more strongly penalized covariates in the model. They also pointed out that the sampling design should be ignorable for, or conditionally independent of, estimating the response given the covariates selected in the final model. Such ignorability conditional on all the covariates would be extremely complicated and beyond researchers’ control [16, 19]. Thus, it is rarely feasible to fit “perfect” ML models with a large number of predictors.

When sampling weights provide extra information in addition to covariates in the prediction model, including sampling weights could improve prediction accuracy. However, as ML algorithms typically include a large number of predictors, this effect might be negligible if important variables had already been included in the model. Our results provide empirical evaluation on effects of incorporating sampling weights in the estimation process for two NCDs. Besides prediction, ML algorithms are often used to identify important predictors for further study. This can also be affected by sampling weights’ inclusion and has not been thoroughly investigated. We also provide empirical observation for ordering important variables by different algorithms with or without sample weights.

Two NCDs presented in this paper also exemplify the class-imbalance problem, often encountered in ML model development; the positive or minority class is insufficiently represented. This problem is not merely common to many real-world applications, such as medical diagnosis, fraud detection, etc. but is considered one of the most challenging problems in ML, data-mining, and knowledge discovery. When fitting standard ML models with class-imbalanced data, ML algorithms suffer from bias toward the majority class , leading to a grave disparity between specificity and sensitivity, simultaneously giving a false impression of high accuracy [20, 21]. Although this is not the theme of the case study, it is likely to be one of the elements to affect the performance of ML models when acting together with sample weight adjustments. To avoid the direct effect on adjusting sample weights to ML models, we balanced the data by two easily applicable under-sampling methods—random under-sampling (RUS) and under-sampling based on clustering (SBC) [22]—before model training. We additionally inspected whether such sampling methods would help alleviate class imbalance when a complex sampling framework determining sample weights was involved in the ML model development. Therefore, the goal of the case study is to explore how sample weights produced by the complex sampling structure affect the performance of prediction on classification by the final ML models depending on the data balancing methods. Due to the aforementioned limitation in implementing ML models with sample weights, we applied replicate weights methods, such as dCV, to Elastic Net (EN), Random Forest (RF) [23], and XGBoost as an extension of R-svyVarSel [16] to facilitate these analyses.

The paper is organized as follows. The Methods section presents the study population of the data, the data processing procedure involving sampling and ML methods, and descriptions on model selection and performance metrics for complex survey data. Results from two examples and the corresponding discussion with future directions are presented in Results, Discussion sections, respectively. The Conclusion section finally remarks the main conclusions.

## 2 Materials and methods

### 2.1 Study population

The NHANES data is sampled by the complex, multi-stage, probability sampling design to examine a nationally representative sample of about 5,000 persons from the civilian, non-institutionalized US population each year. This is a program of studies, consisting of four interviews (demographic, socio-economic, dietary, and health-related questions) and physical examinations (medical, dental, and physiological measurements and laboratory tests), to assess the health and nutritious status of adults and children. Sample weights are created in order to account for the sampling design including oversampling, non-response, and post-stratification adjustment to match the total population size from the Census Bureau. It has thus much less measurement errors than self-reporting health surveys [12, 14]. Because the NHANES program has conducted a series of biennial surveys continuously, the continuity and availability of each variable vary by specific categories and years; some variables are either discontinued due to inconsistent data collection by different cycles or missing as a result from questions conditional on responses of the prior questions. Others are collected by another names in certain cycles. We thus obtained 4 survey cycles of the NHANES data for the two NCDs examples from 2007–2008 to 2013–2014. Based on recent ML-type model developments on hypertension and type 2 diabetes, participants were only selected to be non-pregnant women and adults not less than 20 years old (n=10,891) [11, 24, 25]. Key demographic characteristics of the analytic population after the data pre-processing step are demonstrated in Table 1.

**Table 1:** Key demographic characteristics of analytic population in each dataset.

Variable	Training set	Test set
	(n=6,024)	(n=1,926)
Ratio of family income to poverty${}^{a}$	2.5 (1.6)	2.6 (1.7)
Age group${}^{b}$		
20–39 years	1,924 (31.9%)	590 (30.6%)
40–59 years	2,045 (33.9%)	706 (36.7%)
60+ years	2,055 (34.1%)	630 (32.7%)
Gender		
Male	2,884 (47.9%)	908 (47.1%)
Female	3,140 (52.1%)	1,018 (52.9%)
Ethnicity		
Non-hispanic white	2,838 (47.1%)	894 (46.4%)
Non-hispanic black	1,118 (18.6%)	352 (18.3%)
Mexican American	938 (15.6%)	250 (13.0%)
Other hispanic	661 (11.0%)	177 (9.2 %)
Other hispanic including multi-racial	469 (7.8%)	253 (13.1%)
Education		
Less than 9th grade	666 (11.1%)	122 (6.3%)
9–11th grade (+12th grade without diploma)	883 (14.7%)	260 (13.5%)
High school grad/GED or equivalent	1,369 (22.7%)	403 (20.9%)
Some college or AA degree	1,681 (27.9%)	611 (31.7%)
College graduate or above	1,425 (23.7%)	530 (27.5%)
Marital status		
Never married/separated/divorced/ widowed	2,387 (39.6%)	723 (37.5%)
Married/living with partner	3,637 (60.4%)	1203 (62.5%)
Hypertension		
No	3,796 (63.0%)	1171 (60.8%)
Yes	2,228 (37.0%)	755 (39.2%)
Diabetes		
No	5,298 (87.9%)	1717 (89.1%)
Yes	726 (12.1%)	209 (10.9%)

All participants were filtered by RIDEXPRG = 2 (non-pregnant), RIDAGEYR (age) ≥ 20. Most categorical variables (or factor variables) were rearranged for analysis.

${}^{a}$Mean (standard deviation).

${}^{b}$They were rearranged by the NHANES recommendation.

### 2.2 Data pre-processing

The National Center for Health Statistics (NCHS) in the Centers for Diseases Control and Prevention (CDC) provides full access of the NHANES data to the public. We downloaded the raw data from the database and extracted all candidate variables associated with hypertension and diabetes based on the literature review [11, 24–28]. On the data screening step, we dropped out variables having more than 50% missing values, followed by individuals having more than 10% missing values. We checked the skewness of each factor variable and excluded one if one of the categories or levels accounted for less than 5%. We then had 20 out of 42 variables, all of which included less than 10% missing values but 5 variables: DIABRISK (12.44%), ALQ120Q and ALQ150 (14.3%), HCHOL (14.43%), and ALQ130 (35.24%). They were imputed using the predictive mean matching (pmm) method in a R-mice package [29].

As a rule of thumb, pairwise correlations among independent variables greater than 0.8 indicate the presence of multi-collinearity. We computed correlation coefficients for numeric variables to filter out highly correlated variables. Dropping criteria were followed by Steyerberg’s guidelines [30]: picking easily obtainable one between the two correlated variables, removing one particularly highly correlated with many others, and combining related ones into one variable. For instance, total Kcal of Dietary day one or two (DR1TKCAL,DR2TKCAL) were removed because of the high correlation with fat, protein, sodium, and calcium. Body mass index (BMI) and mean blood pressure (MBP) were computed with weight and height ($\mathrm{kg}/{\mathrm{cm}}^{2}$) and (2DBP+SBP)/3, respectively, where DBP and SBP were short for Diastolic and Systolic blood pressures, respectively. Numeric variables need not be normalized since R-libraries, such as glmnet, xgboost, set normalization to be default. The pre-screened numeric variables are presented by Supplementary Figure S1.

When it came to categorical variables, we recoded levels in ascending order by setting the lowest level to be “0” as a reference category. For example, the questionnaire related to general health conditions, HUQ010 converted 5 levels from excellent (1) to poor (5) into poor (0) to excellent (4). Binary questions recoded “Yes (1)”, “No (2)” to “1”, “0”, respectively. For the correct label assignment of hypertension, we employed the questionnaire, “BPQ020: Ever told you (or your spouse) had hypertension or high blood pressure (HBP) by a doctor?” to label Yes, No as 1, 0, respectively to check it with two variables (BPXSAR/BPXDAR) from the examination data and hypertension category designed by the American Heart Association as systolic (or diastolic) blood pressure of greater than or equal to 130 mmHg or 130/80 [25]. For that of diabetes, we labelled individuals having LBXGLU≥126mg/dL from the lab data as 1 while checking this with a questionnaire for the diagnosis of diabetes by a doctor (DIQ010) [24]. For sample (or sampling) weights, we followed the NHANES guidelines to select the correct weights appropriate for the variables of interest, eliminating non-positive values, i.e., “0” weights. Final candidate variables to conduct modeling are listed on Table 2.

**Table 2:** NHANES: description of candidate variables.

Datasets	Variables${}^{a}$	Variable description
	INDFMPIR	numeric. Ratio of family income to poverty.
Demographic & sample weights	AGEGROUP (RIDAGEYR)	factor.
		Age group relabelled by NHANES recommendation.
	GENDER (RIAGENDR)	factor.
	ETHNICITY (RIDRETH1)	factor.
	EDUC2 (DMDEDUC2)	factor. Education level.
	MARTL (DMDMARTL)	factor. Marital status.
	SEQN	Respondent sequence number.
	SDDSRVYR	Data Release Number (Survey year).
	SDMVPSU	Masked Variance Unit Pseudo-PSU.
	SDMVSTRA	Masked Variance Unit Pseudo-Stratum.
	WTSAF8YR (WTSAF2YR)	Adjusted 4-cycle weights by
		Fasting Subsample 2 Year MEC Weight.
Questionnaire	ALQ101	factor. Had at least 12 alcohol drinks per 1 year?
	ALQ120Q	numeric.
		How often drink alcohol over past 12 months?
	ALQ130	numeric. Avg number of alcoholic drinks
		per day in the past 12 months?
	ALQ150	factor. Ever have 5 or more drinks every day?
	BPQ020	factor. Hypertension or high BP? (Outcome)
	DIABQ (DIQ010)	factor. Diabetes? (Outcome)
	DIABRISK (DIQ170)	factor.
		Ever told have health risk (family history) for diabetes?
	HUQ010	factor. General health condition.
	HUQ020	factor. Health now compared with 1 year ago.
	HUQ050${}^{a}$ (HUQ070)	factor.
		Number of times to receive healthcare over past year.
	HCHOL (BPQ080)	factor. high cholesterol level?
	WHD140	numeric. Self-reported greatest weight (lbs.).
	MCQ300C	factor. Close relative had diabetes?
	PAD680	numeric. Minutes sedentary activity.
	SMOKING${}^{b}$ (SMQ020-040)	factor. Ever smoke 100 cigars in life?
Examination	BMXBMI	numeric. Body Mass Index ($\mathrm{kg}/m^{2}$).
	BMXARML	numeric. Upper Arm Length (cm).
	BMXLEG	numeric. Upper Leg Length (cm).
	BPXPLS	numeric. 60 s pulse (30 s pulse * 2)
	MBP${}^{b}$	numeric. Mean blood pressure.
Dietary${}^{c}$	DR1TPROT/DR2TPROT	numeric. Protein (gm)
	DR1TSUGR/DR2TSUGR	numeric. Total sugars (gm)
	DR1TFIBE/DR2TFIBE	numeric. Dietary fiber (gm)
	DR1TTFAT/DR2TTFAT	numeric. Total fat (gm)
	DR1TCHOL/DR2TCHOL	numeric. Cholesterol (mg)
	DR1TCALC/DR2TCALC	numeric. Calcium (mg)
	DR1TSODI/DR2TSODI	numeric. Sodium (mg)
	DR1TCAFF/DR2TCAFF	numeric. Caffeine (mg)
	DR1TALCO/DR2TALCO	numeric. Alcohol (gm)
Laboratory	LBXGLU	numeric. Fasting Glucose (mg/dL). (Outcome)

All participants were filtered by RIDEXPRG = 2 (non-pregnant), RIDAGEYR (age) ≥ 20.

${}^{a}$All variables in parentheses, if exist, are NHANES-defined variables. They were relabelled for ease of understanding.

${}^{b}$Variables were merged and/or relabelled if any follow-up question exists.

${}^{c}$Prefixes “DR1/DR2” indicate first, second days, respectively.

Sample weights were accordingly tailored to combine adjacent four two-year cycles to produce statistically reliable estimates [12]: they were divided by the number of data cycles, 4. With the adjustment, the combined dataset is considered a simple random sample (SRS). Without such adjustment, we need to assume the whole data to be an independent and identically distributed (iid) sample as unweighted data. We eventually obtained n=6,024, n=1,926 by 43 candidate variables for training and test sets from the first three and the last cycles, respectively, prior to transforming and under-sampling the datasets. All the datasets were matricized and transformed to sparse matrices for efficient computation. R-code for the NHANES data processing with the processed dataset (Supplementary Data—Rcode) are available in Supplementary Material.

### 2.3 Sampling and machine learning methods

Common data level solutions to balance data distribution are over-sampling and under-sampling approaches. They are not merely simple, effective to implement, but sampling process is also independent of learning algorithms. An under-sampling technique is, in particular, popular in that it reduces training time and prevents a model from being overfitted unlike an over-sampling approach. The former approach may lose the representative majority class information; the latter may cause a final model to be overgeneralized by blindly generating synthetic minority class examples. However, any of the methods neither generalizes nor unambiguously yields the outcomes [22, 31–37].

The degree of class imbalance varies with the prevalence of an event, i.e., a disease or condition, so that we have used two useful under-sampling schemes ad hoc to generate balanced datasets: RUS and SBC. The former is a non-heuristic method of eliminating majority class examples at random to balance class distribution [38]; the latter is a method of clustering all samples into K clusters and randomly selecting majority class examples from each cluster to combine with all the minority class examples [22]. Note that NHANES data is sampled by probability proportional to size (PPS) from the target population. The following Lemma [18, 39] supports that a subsample, or a data distribution balanced by the random under-sampling schemes, is the one with the same PPS sampling design as the original sample. It is, moreover, valid for creating folds, i.e., subsamples, for CV in the next subsection.

Lemma 1 Suppose a sample S is a PPS sample with sample size n, drawn from universe U of a known size N. Suppose further that the subsample $S_{m}(\subset S)$ is to be drawn by simple random sampling taking m out of n. Then, $S_{m}$ is a PPS sample with size m, and the second-order inclusion probabilities for distinct pairs of elements of the subsample are also proportional to the corresponding joint inclusion probabilities for the sample S.

As explained earlier, sample weights in the NHANES data are well established. We thus do not consider over-sampling approaches of either re-sampling minority class examples or generating synthetic ones, which leads to reusing sample weights or creating synthetic ones. The RUS was chosen as a baseline method to make a comparison with the SBC for further study. Each under-sampling method produced a pair of balanced training and test sets of n=4,456 and 1,510 for hypertension and of n=1,452 and 418 for diabetes, respectively.

To delve into effects of sample weights on prediction in machine learning, penalized logistic regression [40–42] was utilized for a comparison of results with or without sample weights in the analysis. Since the logistic model for a binary outcome is the most common and is the main interest of this study, we implemented $L_{1}$-logistic regression, i.e., LASSO, as a baseline method to compare with EN and other non-parametric ML methods: RF and XGBoost.

### 2.4 Model selection for complex survey data

In a complex survey framework, sample weights (or sampling weights) are considered when optimal tuning parameters are chosen as well as model parameters are estimated in cross validation. We present a pseudo log-likelihood function with EN penalization, followed by two replicate weights methods for logistic LASSO model’s parameter tuning in R-svyVarSel and extend this to model selection for a weighted logistic EN model.

Let us first define U as a finite population of size N and S(⊂U) as a sample following a complex sampling design, e.g., the NHANES data composed of a stratified four stage probability cluster sample of households. The sampling weight is denoted by $w_{j}=1/\pi_{j}$, where $\pi_{j}=P(j\in S)$ is the sample inclusion probability for unit j (j=1,…,N) computed by the product of all the conditional selection probabilities at corresponding sampling stages. The penalized pseudo log-likelihood function fitting a weighted EN regression model is

$$\min_{\beta }-l_{p}(\beta ;y_{j},x_{j})+\lambda [(1-\alpha )\|\beta {\|}_{2}^{2}+\alpha \|\beta {\|}_{1}],$$ (1)

and the pseudo log-likelihood for logistic regression is

$$l_{p}(\beta ;y_{j},x_{j})=\sum_{\;j\in S}w_{j}\left[y_{j}\ln(p(x_{j}))+(1-y_{j})\ln(1-p(x_{j}))\right],$$ (2)

where $p(x_{j})=1/(1+\exp(-x_{j}\beta ))$ ($\beta =(\beta_{0},\ldots ,\beta_{p}{)}^{\prime }$), $x_{j}$ is the jth vector of p+1 covariates including the intercept, and $y_{j}$ is a binary response variable in $X_{S}=(x_{1},\ldots ,x_{n}{)}^{\prime }$ and $Y_{S}=(y_{1},\ldots ,y_{n}{)}^{\prime }$, respectively [16, 19, 40, 43]. It is noticed that the penalized pseudo log-likelihood function Equation 1, 2 for α=1 is reduced to the one for the weighted logistic LASSO model.

Next, two replicate weights methods are introduced as follows although we have included all the other methods in the package, such as Balanced Repeated Replication (BRR), Rescaling Bootstrap (Bootstrap), Split-sample Repeated Replication (split) including split-cv and split-boot, Extrapolation (extrap). On the basis of the result in [16], BRR, split-cv, and split-boot, and extrap methods selected large tuning parameters resulting in the selection of very few variables while increasing the estimated population error. The Bootstrap method performed best with respect to the error in all scenarios considered; however, the variable selection depends on the scenario. In contrast, the two ensuing methods selected unbiased tuning parameters in terms of the true population parameters, keeping consistently a similar number of variables in the final models.

$S_{te(k)}$ is denoted as a test set of the kth subsample, and the remaining K−1 subsamples make up a training set, $S_{tr(k)}$ in cross validation.

**Jackknife Repeated Replication (JKn)** is typically denoted as leave-one-cluster(or group)-out (LOCO/LOGO) cross validation [44, 45]; for each δ (1≤δ≤Δ) to be a primary sampling unit (PSU), the JKn leaves one PSU as a test set and the remaining Δ−1 PSUs as a training set. For each δ from stratum h (1≤h≤H), replicate weights are defined as follows:

$$w_{\;j,tr(\delta )}^{*}=\left\{ \begin{array}{ccc} 0\; & \text{if }j\in S_{te(\delta )},\;\\ \frac{\delta_{h}}{\delta_{h}-1}w_{j}\; & \text{if }j\in S_{tr(\delta )}\;\;\; & \;\text{in stratum }h,\;\\ w_{j}\; & \text{if }j\in S_{tr(\delta )}\;\;\; & \;\text{not in stratum }h,\; \end{array}\right.$$ (3)

and $w_{\;j,te(\delta )}^{*}=w_{j}$. Denote that $\delta_{h}$ is the number of sampled PSUs in stratum h, and $\sum_{h=1}^{H}\delta_{h}=\Delta$.

**design-based K-fold Cross Validation (dCV)** divides Δ sampled PSUs into K subsets at random, such that the kth subset is a test set while the remaining K−1 subsets form a training set. Then it continues to check if at least one PSU from each stratum exists in every training set, not in every fold, so as for all the PSUs from a stratum not to be in the same fold. In other words, each stratum only requires not less than two PSUs to assort each one of them in a different fold. Replicate weights thus are

$$w_{\;j,tr(k)}^{*}=\left\{ \begin{array}{cc} 0\; & \text{if }j\in S_{te(k)},\;\\ \frac{\delta_{h}}{\delta_{h}-\delta_{h}^{*}}w_{j}\; & \text{if }j\in S_{tr(k)},\; \end{array}\right.$$ (4)

where $\delta_{h}^{*}$ is the number of PSUs from stratum h in $S_{te(k)}$. $w_{\;j,te(k)}^{*}$ is also defined as the same as above by switching the “train” term (*tr*) to the “test” (*te*) [16].

Now, for each sample generated by replicate weights, $S^{r}$ (r=1,…,R), let ${\hat{\;p}}_{r,tr(k)}^{i,m}(x_{j})$ be a predicted value at $j\in S_{te(k)}^{r}$, where ${\hat{\;p}}_{r,tr(k)}^{i,m}(\cdot )$ is fitted with the corresponding training set, $S_{tr(k)}^{r}$ (1≤k≤K), by each pair of tuning parameters ($\alpha_{i}$,$\lambda_{m}$) in Equation 1 on a grid (1≤i≤I, 1≤m≤M). Together with the above methods, one can find a pair of optimal tuning parameters ($\alpha_{i}^{*}$,$\lambda_{m}^{*}$) by minimizing the following estimation error:

$$\min_{\alpha_{i},\lambda_{m}}\frac{1}{RK}\sum_{r=1}^{R}\sum_{k=1}^{K}\frac{1}{\sum_{\;j\in S_{te(k)}^{r}}w_{j}^{r}}\sum_{\;j\in S_{te(k)}^{r}}w_{j}^{r}L(y_{j},{\hat{\;p}}_{r,tr(k)}^{i,m}(x_{j})),$$ (5)

where the loss function for logistic regression is

$$L(y_{j},{\hat{\;p}}_{tr(k)}^{i,m}(x_{j}))=-y_{j}\ln({\hat{\;p}}_{tr(k)}^{i,m}(x_{j}))-(1-y_{j})\ln(1-{\hat{\;p}}_{tr(k)}^{i,m}(x_{j})).$$ (6)

Note that the dCV (Equation 4–6) is the counterpart of the traditional K-fold CV for complex survey data and is thus the main concern of this study. The JKn (Equation 3, 5, 6) is rigorous, computationally demanding, and thus impractical for computers with limited memory capacity. In terms of non-parametric algorithms, we only consider the dCV for XGBoost, due to the high computational demand in hyperparameter tuning in the model selection process. However, we consider both dCV and JKn for RF since RF is the only package including the option of managing a stratified sampling framework so is worthy of investigating the usefulness.

### 2.5 Performance metrics

We initially assessed the performance of ML models by measuring the following performance metrics: a ROC curve, and its AUC, accuracy, sensitivity (or recall), specificity. Since the purpose of this study is how sample weights accounting for a complex survey framework influence final models’ performance, it is not sufficient to measure these standard performance metrics from the confusion matrix by simply comparing outcomes with corresponding predicted values from the final weighted ML models. We employed the weighted performance metrics as follows: a weighted ROC (wROC) curve, its weighted AUC (wAUC), weighted-sensitivity (wSensitivity), and weighted-specificity (wSpecificity). Since complex survey data are contingent on the proportion of sampled units from the corresponding strata, unweighted estimators for the ROC curve and AUC either overestimate or underestimate the true population parameters. The wROC and wAUC, however, show slight overestimation to the true population parameters [46], which we have observed to be more negligible than that by their unweighted counterparts. We have thus taken advantage of these metrics for evaluating the final weighted ML models, assuming that a wROC curve and its wAUC estimate the true population parameters. wSensitivity, wSpecificity can be obtained by estimating an optimal cutoff of the probability of an event of interest (or a disease) with sample weights by maximizing a Youden index, defined by subtracting 1 from the sum of sensitivity and specificity. The Youden method is one of the most common estimation methods in the literature and is used to compute a ROC curve, incorporated with the ROC01 method in R-pROC [47–49] as well. When multiple optimal cutoffs were estimated, we picked the one with the highest wSensitivity. We also computed bootstrap 95% confidence intervals (CIs) of AUC, wAUC values for the final ML models selected by the K-fold CV and dCV, respectively using R-pROC and R-svyROC [46]; jackknife 95% CIs of the wAUC were done by replacing the bootstrap method with the JKn accordingly.

### 2.6 Overall process

To compare final weighted ML models to corresponding unweighted ones estimated by the same data sets, we divided training and test sets by survey cycle: 2007–2012 (3 cycles) for the training set and 2013–2014 (1 cycle) for the test set after the data preprocessing step. We generated two pairs of balanced training and test sets for each example by the under-sampling methods: n=4,456 and 1,510 for hypertension and n=1,452 and 418 for diabetes. The severity of class imbalance in diabetes, whose ratio of the majority class (non-disease or 0) to the minority (disease or 1) was about 7.5 to 1, yielded much smaller balanced data sets than those for hypertension, whose ratio was about 1.63 to 1.

We then proceeded to train four ML models to select the best model for each ML method on the CV step; for unweighted data-driven models, the (traditional) K-fold CV was carried out while the K-fold dCV incorporated with R replicate sample weights and Jkn were performed for weighted ones. CV methods were performed with K=10 folds and R=10 replicate weights. For instance, the 10-fold dCV per replicate weights was implemented in the weighted model development. For EN, we employed R-glmnet and glmnetUtil [40–42] to search for an optimal set of hyperparameters ($\alpha^{*},\lambda^{*}$) for unweighted models during the CV procedure for each disease outcome. R-MLSurvey has included this grid search for weighted EN models with replicate weights methods as well. Note that using a CV function for EN, “cva.glmnet” yields graphs of log⁡λ corresponding to grids of α, showing that α=0.729 gives rise to the second best optimal plot since the best one is α=1 (LASSO, or $L_{1}$). RF need not carry out the K-fold CV for simple random samples, or unweighted data, to avoid overfitting since it finds optimal tuning parameters with default initial values without CV [23]. We have also confirmed this by comparing with “tuneRF” function for optimal hyperparmeter selection, showing the former is more optimal than the latter. We only implemented RF with replicate weights methods in the weighted model development. For XGBoost, hyperparameter optimization was rigorously performed before training models for both models’ development. We randomly selected a set of hyperparmeters (“max_depth”,“eta”,“gamma”,“subsample”,“colsample_bytree”) with “seed number” to run “xgb.cv” to find the best iteration number for each iteration and then chose the best set to train the model for unweighted models. Final weighted models were also selected by the same procedure with a “wXGBoost” function in our R-package.

Each selected model on the validation step was evaluated on the test set by performance metrics. 2000 bootstrap replicates were generated to compute 95% CIs for wAUC values of the final models by the 10-fold dCV and AUC values by the 10-fold CV, respectively. Jackknife 95% CIs were computed for wAUC estimates. Note that (unweighted) standard performance metrics, i.e., accuracy, sensitivity, and specificity, were computed by R-caret [50], where they were measured by the confusion matrix without sample weights. All work was carried out in R v.4.6.0 [51]; R-code scripts for the detailed implementation of data analysis are available in Supplementary Material.

## 3 Results

Tables 3, 4 display overall performance on the final ML models for two NCDs. Weighted performance metrics are presented adjacent to the (unweighted) standard performance metrics depending on the sampling methods to observe how unweighted metrics are deviated from the weighted ones. Since wAUC and AUC are approximated by different methods, we have validated their equality provided that no information on sampling design other than covariates was included, i.e., no sample weights in the model. Tables 3, 4 demonstrate that wAUC values and corresponding CIs are equal to the counterparts for unweighted ML models, i.e., AUC=wAUC. One can simply pay attention to wAUC values to compare the performance between a final unweighted and the corresponding weighted models for each ML method. Notice that the diabetes example reveals only the subtlety between these CIs due to estimation methods combined with the severity of class imbalance. For the final weighted models, we have also computed AUC values (unweighted values), showing to be higher or lower than their weighted counterparts, or wAUC values. This implies the AUC of the weighted ML model is biased. wSensitivity and wSpecificity suggest that the standard performance metrics obtained from the confusion matrix should be optimally corrected by sample weights.

**Table 3:** Hypertension: performance of final unweighted and weighted ML models depending on the under-sampled data.

ML model	Method	Sampling	AUC	wAUC	Accuracy	Sensitivity	wSen${}^{a}$	Specificity	wSpec${}^{a}$
			(95% CI)	(95% CI)					
Logistic Reg.-$L_{1}$ (LASSO)	CV	RUS	0.838	0.838	0.756	0.766	0.815	0.747	0.719
			(0.817, 0.857)	(0.818, 0.857)					
		SBC	0.846	0.846	0.771	0.772	0.803	0.770	0.743
			(0.825, 0.865)	(0.826, 0.866)					
	dCV	RUS	0.833	0.829	0.758	0.762	0.719	0.754	0.785
			(0.813, 0.852)	(0.804, 0.854)					
		SBC	0.844	0.826	0.763	0.736	0.724	0.789	0.772
			(0.825, 0.862)	(0.804, 0.848)					
	JKn	RUS	0.833	0.829	0.760	0.764	0.723	0.755	0.784
			(0.833, 0.834)	(0.824, 0.835)					
		SBC	0.845	0.826	0.764	0.742	0.722	0.785	0.773
			(0.845, 0.846)	(0.821, 0.831)					
Logistic Reg.- EN (α=0.729)	CV	RUS	0.838	0.838	0.757	0.766	0.820	0.748	0.713
			(0.817, 0.857)	(0.825, 0.865)					
		SBC	0.844	0.844	0.77	0.771	0.776	0.770	0.770
			(0.824, 0.864)	(0.825, 0.864)					
	dCV	RUS	0.833	0.83	0.759	0.760	0.783	0.758	0.722
			(0.813, 0.852)	(0.804, 0.854)					
		SBC	0.844	0.826	0.762	0.735	0.710	0.788	0.787
			(0.825, 0.863)	(0.804, 0.848)					
	JKn	RUS	0.833	0.83	0.76	0.764	0.785	0.756	0.721
			(0.833, 0.834)	(0.824, 0.835)					
		SBC	0.845	0.826	0.762	0.738	0.716	0.785	0.780
			(0.844, 0.845)	(0.820, 0.831)					
XGBoost	CV	RUS	0.829	0.829	0.717	0.721	0.804	0.714	0.699
			(0.809, 0.849)	(0.808, 0.848)					
		SBC	0.837	0.837	0.742	0.764	0.824	0.719	0.697
			(0.816, 0.857)	(0.816, 0.856)					
	dCV	RUS	0.807	0.795	0.717	0.725	0.772	0.710	0.379
			(0.786, 0.828)	(0.770, 0.820)					
		SBC	0.812	0.797	0.725	0.701	0.779	0.748	0.694
			(0.790, 0.834)	(0.771, 0.825)					
RF	CV	RUS	0.823	0.823	0.732	0.766	0.819	0.699	0.678
			(0.802, 0.843)	(0.801, 0.843)					
	(unwRF${}^{b}$)	SBC	0.837	0.837	0.751	0.809	0.801	0.693	0.715
			(0.817, 0.857)	(0.817, 0.857)					
	CV	RUS	0.819	0.817	0.743	0.762	0.777	0.725	0.458
			(0.798, 0.839)	(0.799, 0.835)					
	(wRF${}^{b}$)	SBC	0.834	0.82	0.760	0.807	0.811	0.713	0.679
			(0.814, 0.854)	(0.806, 0.834)					
	dCV	RUS	0.822	0.821	0.738	0.752	0.773	0.723	0.712
			(0.801, 0.843)	(0.797, 0.845)					
		SBC	0.836	0.822	0.758	0.799	0.819	0.717	0.663
			(0.816, 0.856)	(0.800, 0.846)					
	JKn	RUS	0.824	0.82	0.741	0.755	0.792	0.727	0.692
			(0.823, 0.824)	(0.815, 0.826)					
		SBC	0.833	0.82	0.758	0.805	0.834	0.711	0.670
			(0.833, 0.834)	(0.814, 0.825)					

All values were rounded off to three decimal places.

${}^{a}$wSen: wSensitivity, wSpec: wSpecificity. For a case yielding multiple optimal cutoffs, the one corresponding to the highest wSensitivity was picked.

${}^{b}$unwRF: a unweighted model, wRF: a weighted model adjusted by the built-in “strata” option in R-randomForest.

**Table 4:** Diabetes: performance of final unweighted and weighted ML models depending on the under-sampled data.

ML model	Method	Sampling	AUC	wAUC	Accuracy	Sensitivity	wSen${}^{a}$	Specificity	wSpec${}^{a}$
			(95% CI)	(95% CI)					
Logistic Reg.-$L_{1}$ (LASSO)	CV	RUS	0.84	0.84	0.763	0.799	0.799	0.727	0.761
			(0.803, 0.875)	(0.801, 0.877)					
		SBC	0.866	0.866	0.773	0.804	0.789	0.742	0.823
			(0.833, 0.900)	(0.830, 0.899)					
	dCV	RUS	0.831	0.841	0.754	0.770	0.729	0.737	0.842
			(0.791, 0.867)	(0.804, 0.877)					
		SBC	0.863	0.852	0.789	0.751	0.774	0.828	0.819
			(0.825, 0.896)	(0.811, 0.894)					
	JKn	RUS	0.831	0.841	0.758	0.775	0.729	0.742	0.833
			(0.830, 0.833)	(0.833, 0.850)					
		SBC	0.864	0.851	0.789	0.751	0.720	0.828	0.858
			(0.863, 0.867)	(0.840, 0.862)					
Logistic Reg.- EN (α=0.729)	CV	RUS	0.84	0.84	0.751	0.775	0.794	0.727	0.775
			(0.803, 0.875)	(0.801, 0.876)					
		SBC	0.857	0.857	0.77	0.804	0.789	0.737	0.823
			(0.833, 0.900)	(0.830, 0.899)					
	dCV	RUS	0.831	0.842	0.756	0.775	0.729	0.737	0.842
			(0.791, 0.867)	(0.806, 0.878)					
		SBC	0.862	0.851	0.787	0.746	0.720	0.828	0.855
			(0.825, 0.895)	(0.811, 0.893)					
	JKn	RUS	0.831	0.842	0.756	0.775	0.729	0.737	0.842
			(0.830, 0.833)	(0.834, 0.851)					
		SBC	0.863	0.850	0.787	0.746	0.720	0.828	0.858
			(0.862, 0.866)	(0.839, 0.862)					
XGBoost	CV	RUS	0.82	0.82	0.746	0.804	0.78	0.689	0.756
			(0.780, 0.858)	(0.779, 0.860)					
		SBC	0.857	0.857	0.77	0.809	0.789	0.732	0.804
			(0.819, 0.893)	(0.819, 0.890)					
	dCV	RUS	0.78	0.796	0.708	0.722	0.746	0.694	0.716
			(0.736, 0.821)	(0.744, 0.843)					
		SBC	0.809	0.814	0.722	0.622	0.816	0.823	0.695
			(0.766, 0.849)	(0.764, 0.862)					
RF	CV	RUS	0.828	0.828	0.749	0.813	0.866	0.684	0.660
			(0.790, 0.866)	(0.787, 0.864)					
	(unwRF${}^{b}$)	SBC	0.859	0.859	0.778	0.856	0.799	0.699	0.775
			(0.821, 0.893)	(0.823, 0.894)					
	CV	RUS	0.822	0.834	0.758	0.780	0.873	0.737	0.673
			(0.781, 0.861)	(0.808, 0.860)					
	(wRF${}^{b}$)	SBC	0.851	0.851	0.78	0.732	0.643	0.828	0.907
			(0.813, 0.888)	(0.818, 0.881)					
	dCV	RUS	0.817	0.83	0.749	0.770	0.859	0.727	0.710
			(0.776, 0.857)	(0.795, 0.864)					
		SBC	0.845	0.847	0.756	0.727	0.824	0.785	0.746
			(0.806, 0.881)	(0.806, 0.887)					
	JKn	RUS	0.818	0.828	0.746	0.751	0.819	0.742	0.734
			(0.817, 0.821)	(0.821, 0.835)					
		SBC	0.851	0.853	0.78	0.742	0.766	0.818	0.785
			(0.850, 0.854)	(0.841, 0.864)					

All values were rounded off to three decimal places.

${}^{a}$wSen: wSensitivity, wSpec: wSpecificity. For a case yielding multiple optimal cutoffs, the one corresponding to the highest wSensitivity was picked.

${}^{b}$unwRF: a unweighted model, wRF: a weighted model adjusted by the built-in “strata” option in R-randomForest.

### 3.1 Hypertension

**Figure 1:**
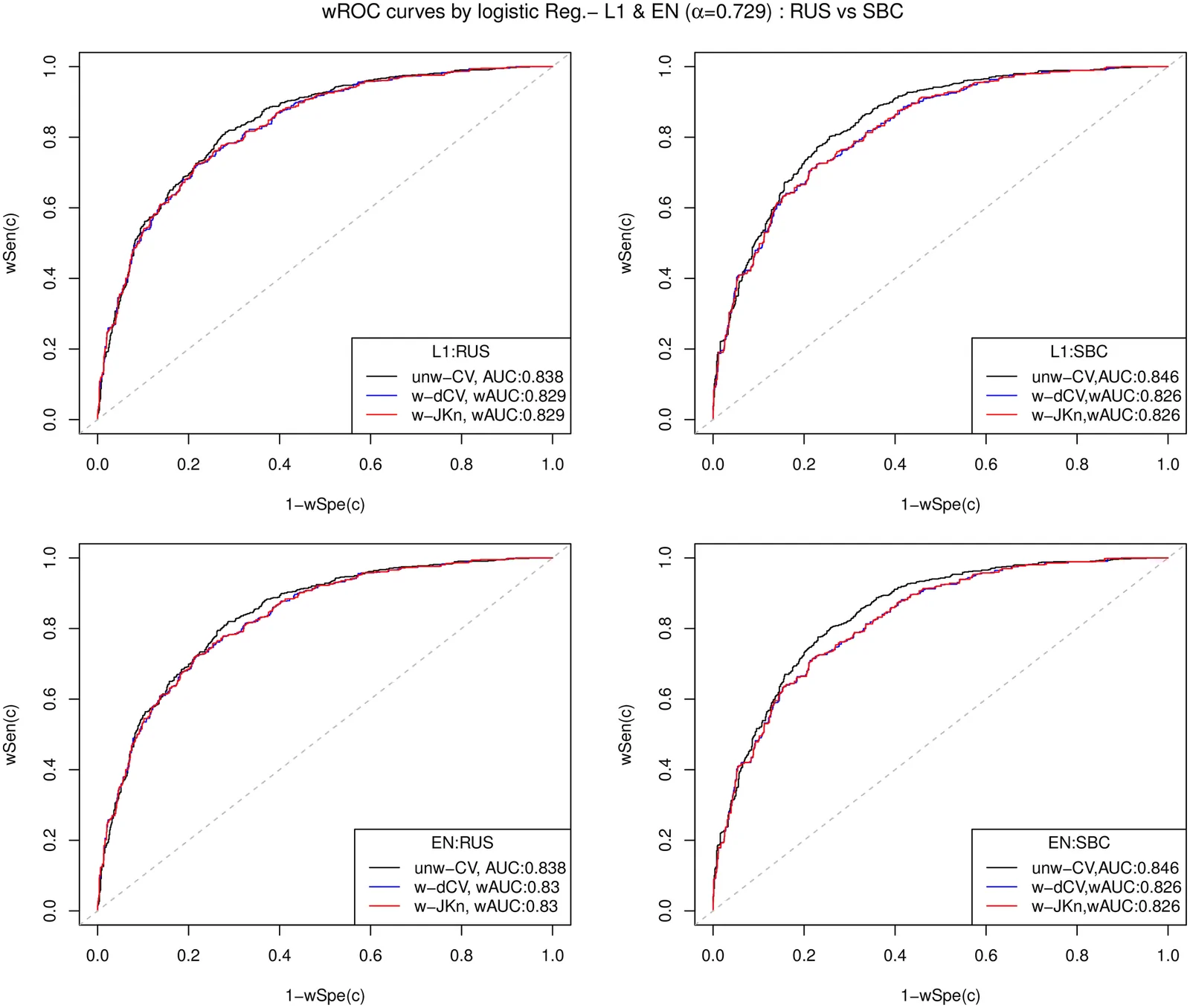
Hypertension: ROC and wROC curves (RUS vs. SBC) by Logistic Reg.-$L_{1}$, EN (α=0.729). wROC curves for unweighted models labelled as “unw-CV” are ROC curves, i.e., AUC=wAUC.

**Figure 2:**
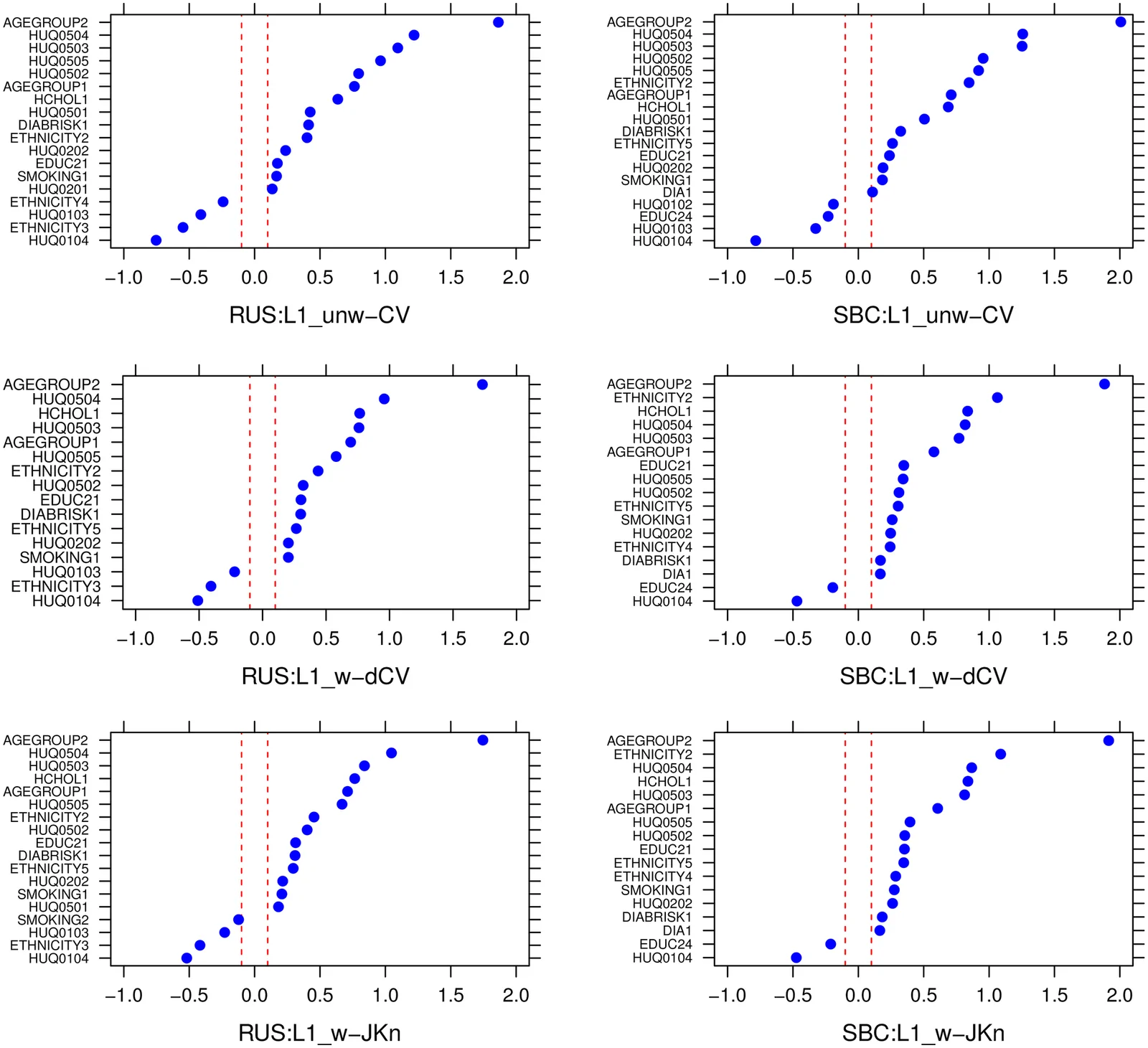
Hypertension: variable selection (RUS vs. SBC) by Logistic Reg.-$L_{1}$. Unweighted and weighted models are labelled as “$L_{1}$_unw” and “$L_{1}$_w”, respectively.

**Parametric algorithms (L1 and EN (α=0.729))**: As shown in Table 3 and Figure 1, AUC (or wAUC) values of the unweighted models are higher than wAUC values of the weighted; AUC measures higher scores than wAUC. We have additionally examined how sampling methods as well as sample weight adjustments determine variable selection. As shown in [16], we have observed that variable selection by the dCV and JKn leads to more parsimonious models than that by the CV (Table 5). In other words, larger tuning parameters (λ’s) are chosen for the weighted models than those for the unweighted. Figure 2 further suggests that the effects size is affected by the sample weights, so as for selected variables to be realigned accordingly. Note that we have excluded variables with small effects between −0.1 and 0.1, considering less important although further tests may need to determine the statistical significance. Variable selection from both sampling methods shows the dCV and JKn select variables having large effects similarly but select ones having smaller effects near ±0.1 a little differently. We have observed that the dCV often selects less variables than the JKn for both $L_{1}$ and EN.

**Table 5:** Hypertension: variable selection and optimal tuning parameters for Logistic Reg.

	$L_{1}$				EN (α=0.729)			
	log⁡λ		# of variables${}^{a}$		log⁡λ		# of variables${}^{a}$	
Methods	RUS	SBC	RUS	SBC	RUS	SBC	RUS	SBC
CV	−5.75	−6.07	36	45	−5.62	−5.84	39	48
dCV	−5.39	−5.24	33	35	−5.17	−5.02	34	36
JKn	−5.58	−5.42	34	36	−5.26	−5.11	35	36

Variables were optimally selected by corresponding methods based on the sparse matrices of candidate variables. The 10-fold CV was implemented for the unweighted data. The 10-fold dCV for 10 replicate sample weights and JKn were implemented for the weighted data.

${}^{a}$The number of variables selected on the cross-validation step.

**Figure 3:**
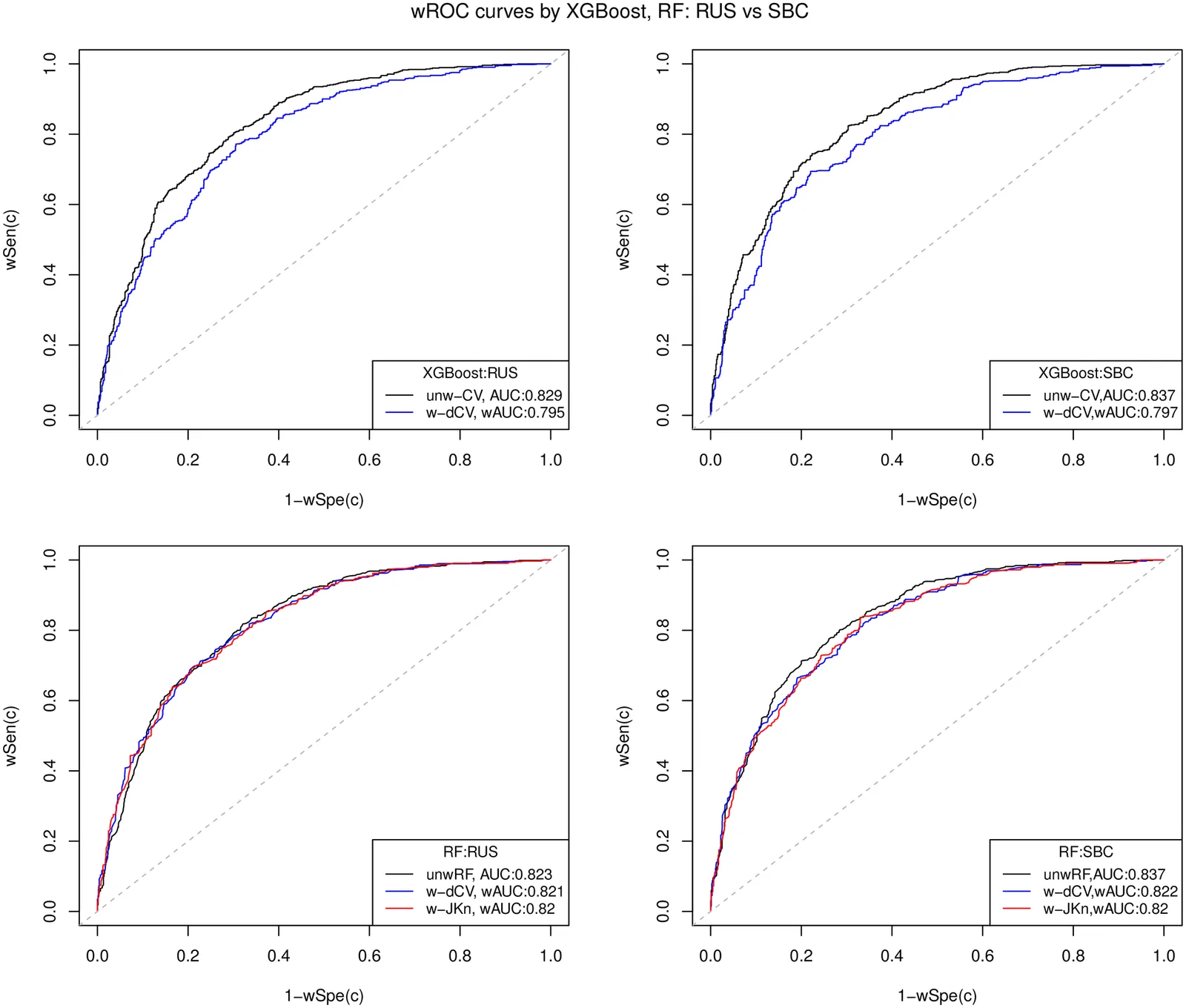
Hypertension: ROC and wROC curves (RUS vs. SBC) by XGBoost, RF. wROC curves for unweighted models labelled as “unw-CV” are ROC curves, i.e., AUC=wAUC.

**Figure 4:**
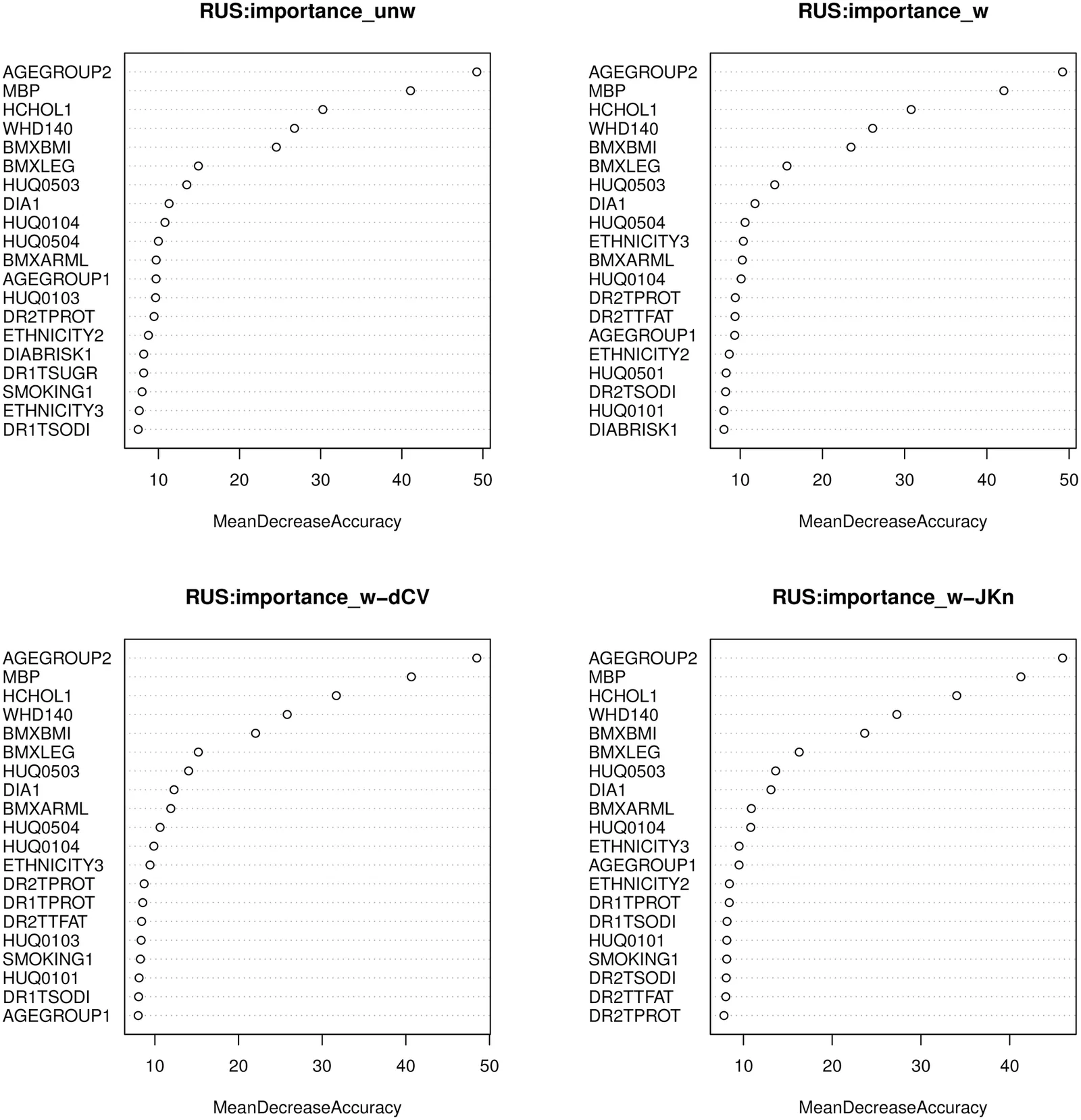
Hypertension: variable selection (RUS) by RF. “importance_unw” is modelled by unwRF and “importance_w” is modelled by wRF with the strata option.

**Figure 5:**
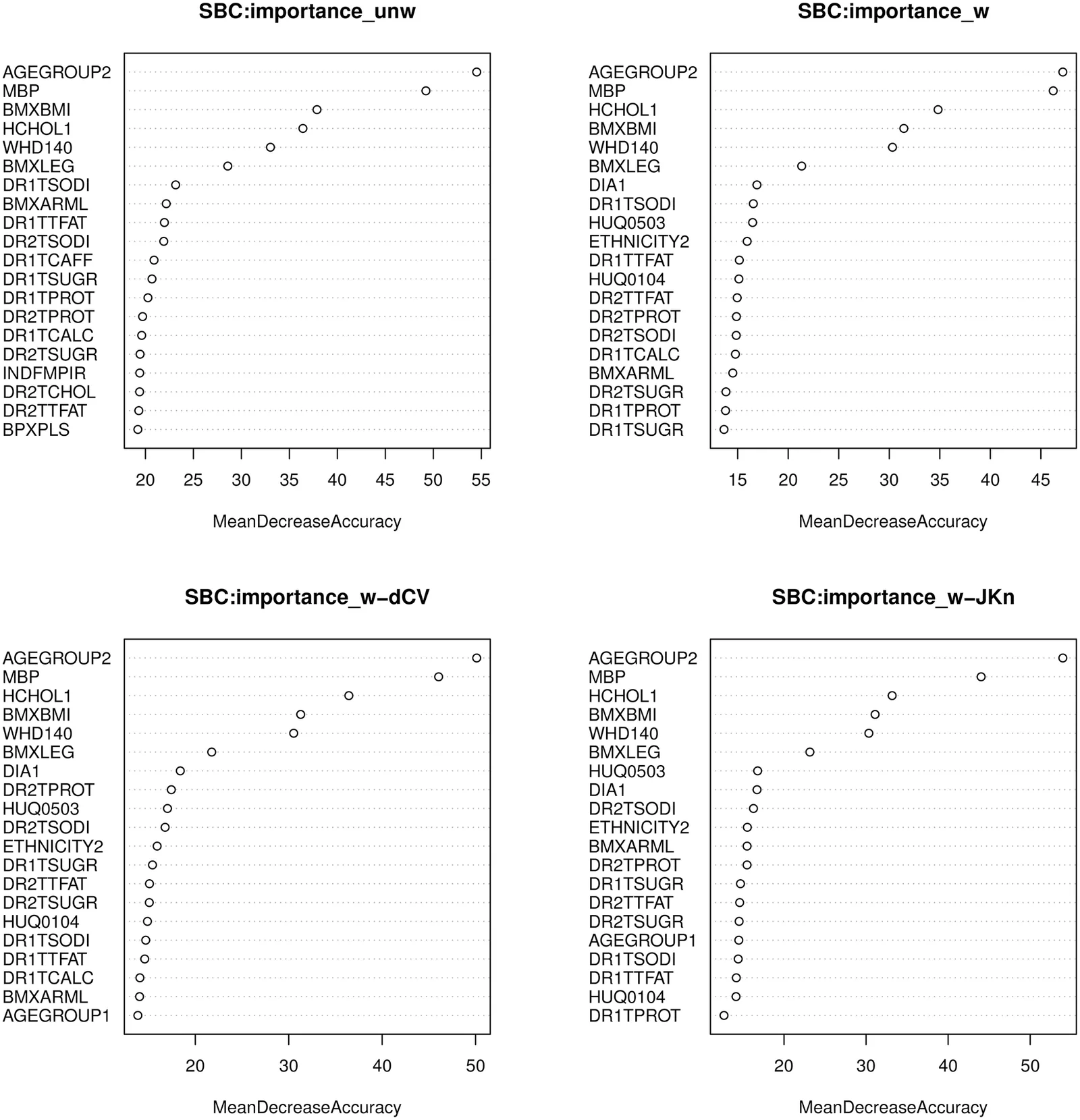
Hypertension: variable selection (SBC) by RF. “importance_unw” is modelled by unwRF and “importance_w” is modelled by wRF with the strata option.

**Figure 6:**
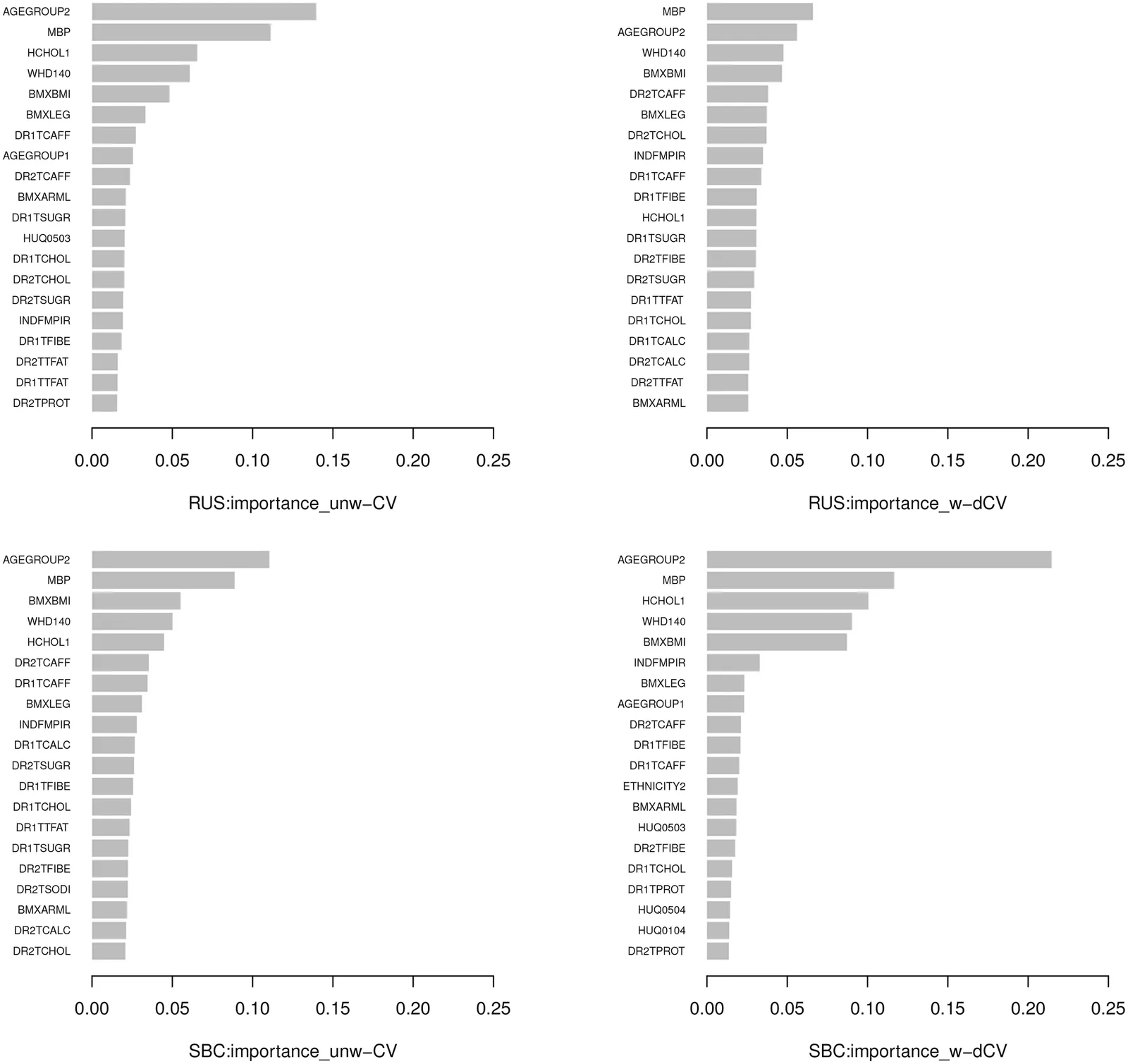
Hypertension: variable selection (RUS vs. SBC) by XGBoost.

**Non-parametric algorithms (RF, XGBoost)**: Note that the R-randomForest, has an option of “strata” for stratified sampling along with sample weights, “weights”. To see how this option works, we included a weighted model fitted by the option to compare a unweighted model with weighted models: unwRF vs. wRF (“strata” option), dCV, and JKn. Both ML methods demonstrate that AUC values are higher than wAUC ones in Figure 3 and Table 3. Moreover, variable selection for RF differs by the sampling methods according to variable importance plots in Figures 4, 5. Comparing unwRF with the others suggests that selected variables within about top 6 be the same by and large with minor difference in order, which may be caused by either bagging or sample weights. It is implied that lower scored variables appear to be more susceptible to variable selection due to the sample weights’ correction. Meanwhile, variable importance plots (Figure 6) for XGBoost show only a few of high scored variables are consistently selected depending on the sample weights’ inclusion and sampling methods. This implies that XGBoost is more influenced by adjusting sample weights to the data than RF.

### 3.2 Diabetes

**Parametric algorithms (L1 and EN (α=0.729))**: As in Table 4 and Supplementary Figure S2, AUC values of unweighted models are either higher or lower than wAUC values of weighted models depending on the sampling methods. It is noteworthy that the K-fold dCV, JKn select more variables than the K-fold CV for both under-sampled data; that is, smaller λ’s in most cases are chosen for the final weighted models than those for the unweighted (Table 6), which is not in accord with the result shown in [16]. Effects size plots excluding small effects between ±0.1 in Supplementary Figure S3 display the impact of sample weights on effects size; selected variables differ by the sampling methods.

**Table 6:** Diabetes: variable selection and optimal tuning parameters for Logistic Reg.

	$L_{1}$				EN (α=0.729)			
	log⁡λ		# of variables${}^{a}$		log⁡λ		# of variables${}^{a}$	
Methods	RUS	SBC	RUS	SBC	RUS	SBC	RUS	SBC
CV	−5.43	−5.52	46	44	−5.11	−5.29	46	46
dCV	−5.50	−5.15	52	46	−5.18	−5.11	51	48
JKn	−5.41	−5.61	50	48	−5.18	−5.30	51	48

Variables were optimally selected by corresponding methods based on the sparse matrices of candidate variables. The 10-fold CV was implemented for the unweighted data. The 10-fold dCV for 10 replicate sample weights and JKn were implemented for the weighted data.

${}^{a}$The number of variables selected on the cross-validation step.

**Non-parametric algorithms (RF, XGBoost)**: Unlike the hypertension example, The AUC value of the unweighted model from RF modelled with the RUS-balanced data only shows to be lower than or equal to wAUC values of the weighted models (Table 4, Supplementary Figure S4). RF demonstrates only subtle differences among wAUC values for each sampling method as well. Variable importance plots for RF (Supplementary Figures S5, S6) reveal more distinction in variable importance than those presented in the hypertension example depending on not merely sample weights’ adjustment but also sampling methods. Nevertheless, among model selection methods that account for a complex sampling framework, e.g., wRF, dCV, and JKn, shows that major importance variables are selected consistently while minor ones are selected irregularly just as in the hypertension. Meanwhile, variable importance plots for XGBoost (Supplementary Figure S7) are noticeable in order due to sample weights’ inclusion in conjunction with the sampling methods.

## 4 Discussion

We have delved into the effect of sample weights on the performance of final ML models relying on the two under-sampling methods: RUS, SBC. They are data level approaches to relieve class imbalance. Four ML models, i.e., logistic regression with $L_{1}$ and EN, RF, and XGBoost, have been fitted with the two balanced NCD data extracted from NHANES: hypertension and diabetes. For the unweighted data-driven model development, variables were selected by the K-fold CV whereas for the weighted, complex sampling design variables composed of PSUs (or clusters), strata, and sample weights were incorporated into the CV process via replicate weights, such as dCV, JKn. We applied the replicate weights methods to logistic regression with EN penalization, RF, and XGBoost for variable selection. The R-package is available on github at https://github.com/hkim-fda/MLSurvey. The weighted performance metrics, i.e., wROC, wAUC, wSensitivity, and wSpecificity, as well as the standard performance metrics have been utilized to check the performance of the final weighted models in comparison with the unweighted. We have first verified that wAUC values and the corresponding CIs are equal to AUC values and the CIs, respectively, for the unweighted ML models. The AUC is off the mark from the wAUC when estimated from the weighted models.

In conjunction with the aforementioned wAUC and AUC, accuracy measured by the confusion matrix is overall similar between weighted and unweighted models. This suggests that including sampling weights does not provide much more information on the population than including only covariates in the ML models does. This may be because the dataset collected for hypertension and diabetes, where extensive literature exists, would be rich in information on the population. The final ML models thus are very likely to include a comprehensive set of covariates. In this case, using weights may cause a slight loss of efficiency and may not be critical for prediction accuracy, as well as the AUC criterion. For less studied diseases, however, sampling weights may provide additional information when a set of covariates is not as complete as that of the two NCDs. Prediction accuracy is not the only consideration in the ML model development; ordering important variables is often of interest in data analysis.

The results show a noticeable difference caused by the degree of class imbalance in the case study. The hypertension example, considered rather class-balanced, shows consistent results with those from the weighted logistic LASSO regression method in [16]. Both penalized logistic regression methods ($L_{1}$, EN) embodying the complex sampling design variables fit more parsimonious models than those without the design variables. The diabetes example, considered a prototype of severe class imbalance, yields contrary results to the hypertension example. The penalized logistic regression algorithms fit more complex models when incorporating with sample weights, or the design variables. The final unweighted models, according to the wAUC, either overestimate or underestimate the true population parameters in most cases but rarely approximate the true population parameters depending on random resampling, ML algorithms, or replicate weights methods as shown from the RF model by JKn and RUS in Table 4. The unweighted RF models in comparison to the weighted depending on the two sampling methods, overall, show consistency in variable selection as well as in the performance. One can still observe that fewer important variables in the diabetes example than those in the hypertension example are selected consistently; yet, the order of importance is much influenced by the sample weights’ inclusion for each sampling method. In contrast, XGBoost may be a good choice for unweighted model development, i.e., SRS or typical iid data; the choice of sampling methods due to the degree of class imbalance yields conspicuously inconsistent results together with sample weights’ adjustment.

Although the two examples in this case study may not be sufficient to generalize our results, they provide us with insight into complex survey data pertaining to class imbalance problems even after reducing class imbalance ad hoc in aspects of both non-parametric ML algorithms. The performance of RF and XGBoost leads to diverse results on account of the underlying algorithmic property: the difference between bagging and boosting. RF appears to weaken the effect of sample weights on the performance among unweighted and weighted models for each sampling method since there exist only minor differences among performance metrics as well as in variable selection although severe class imbalance somewhat fluctuates the overall result. On the contrary, XGBoost does impact variable selection and the final model performance depending on the complex sampling design inclusion into the model with the different sampling methods. This may be caused by the nature of boosting that weights more to weak learners for better detection; it is unknown which data points, selected by random iid sampling to balance data distribution, will be more weighted or not. Then, weighting to weak learners which may have more sample weights or less would be responsible for far worse or better performance, leading to inconsistent results in complex survey data analysis.

For the two disease examples from the NHANES data, the effect of sampling weights on the predictive performance is moderate though varying by algorithm and class imbalance. However, sample weights could provide valuable information on predictive performance and model interpretation if the covariate set were limited or there existed missing regressors. There has been extensive discussion in the survey sampling literature regarding practices of sampling weights to the standard regression setting, which is also applicable, in principle, to machine learning models with an incomplete covariate set [52–54]. Thus, practitioners should ideally take into account sampling weights in their model development and may consider fitting both weighted and unweighted models to detect potentially hidden biases. At the very least, evaluating models with the weighted performance metrics (wAUC, wSpecificity, and wSensitivity) would provide an alert on the potentially incorrect impression of actual model performance.

Class-imbalance is ubiquitous just as this case study reveals; our weighted ML methods (R-MLSurvey) offer unbiased model estimation for complex survey data by ML methods and extended use of ML model prediction to complex survey data for broader research disciplines in need of sufficiently large data including, but not limited to, public health, biomedical research, and social (behavioral) science. We have additionally observed that the aforementioned pattern of variable selection is not always true, perhaps owing to random sampling methods to mitigate class imbalance; even if larger λ were chosen by the K-fold dCV and JKn than by the K-fold CV, slightly more variables would be selected from time to time (Table 6). This is more often observed in the diabetes example. For broader adoption of weighted ML methods, the future research extends to (Deep) Neural Network models for high-dimensional complex survey data; a contrastive variational autoencoder would be adapted to under-sampling methods to remedy class imbalance, preserving the latent data structure with minimal loss of information, with or without sample weights. Finally, adding model-agnostic interpretation tools, such as partial dependence plots (PDP) or SHAP-based explanation, to our R package will be able to improve weighted ML model interpretability.

## 5 Conclusion

We have scrutinized the effect of sample weights associated with a complex survey data structure on the prediction of four ML models depending on two under-sampling methods for relieving class imbalance. The sampling design seems not to furnish significant additional information on prediction accuracy for these relatively well-studied diseases; however, ordering the most important variables is notably affected by incorporating sample weights, governed by the sampling design, into the model development depending on the degree of class imbalance and ML algorithms. We recommend carrying out ML model selection with care when one is working with class-imbalanced data particularly collected by complex sampling design to avoid biased results. Under the limited option of existing ML software packages accounting for such sample weights, RF can be implemented following ad hoc under-sampling methods. However, we emphasize that this proposed approach will not be appropriate for extremal events, such as, rare diseases. Our extension of replicate weights to EN, RF, and XGBoost can facilitate data analysis using complex survey data or in need of sufficiently big data to take advantage of complex survey data for unbiased estimation. The future directions for software development will benefit wider adoption in research communities as well.

## Data Availability

Publicly available datasets were analyzed in this study. This data can be found here: NHANES, https://wwwn.cdc.gov/nchs/nhanes/. The supplementary material including R-code scripts with the final cleaned data and its analyses, figures are available.
