# Supplementary material for: Machine learning models for chronic disease risks using complex survey data: the impact of sample weights
Source: Front Digit Health. 2026 Aug 10;8:1834715. doi: 10.3389/fdgth.2026.1834715 (PMC13501523; doi:10.3389/fdgth.2026.1834715)
Supplement: Supplementary file 1 [file Datasheet1.pdf]

Supplementary Material

1 SUPPLEMENTARY DATA

Supplementary R-scripts for generating the cleansed final data and analyses are provided in **Rcode** folder.

2 SUPPLEMENTARY FIGURES

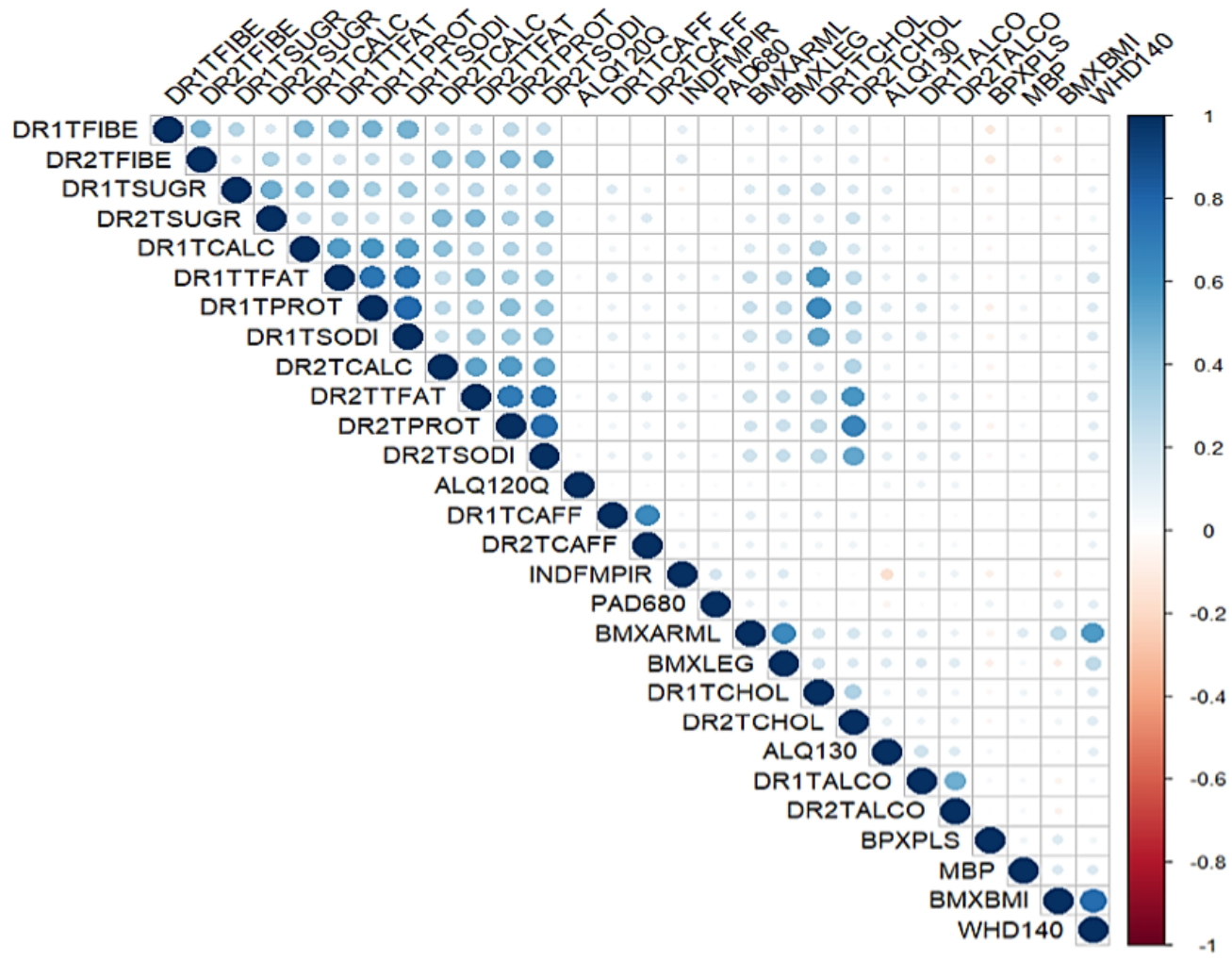

Figure S1. Correlation coefficients of candidate numeric variables selected on the data preprocessing step.

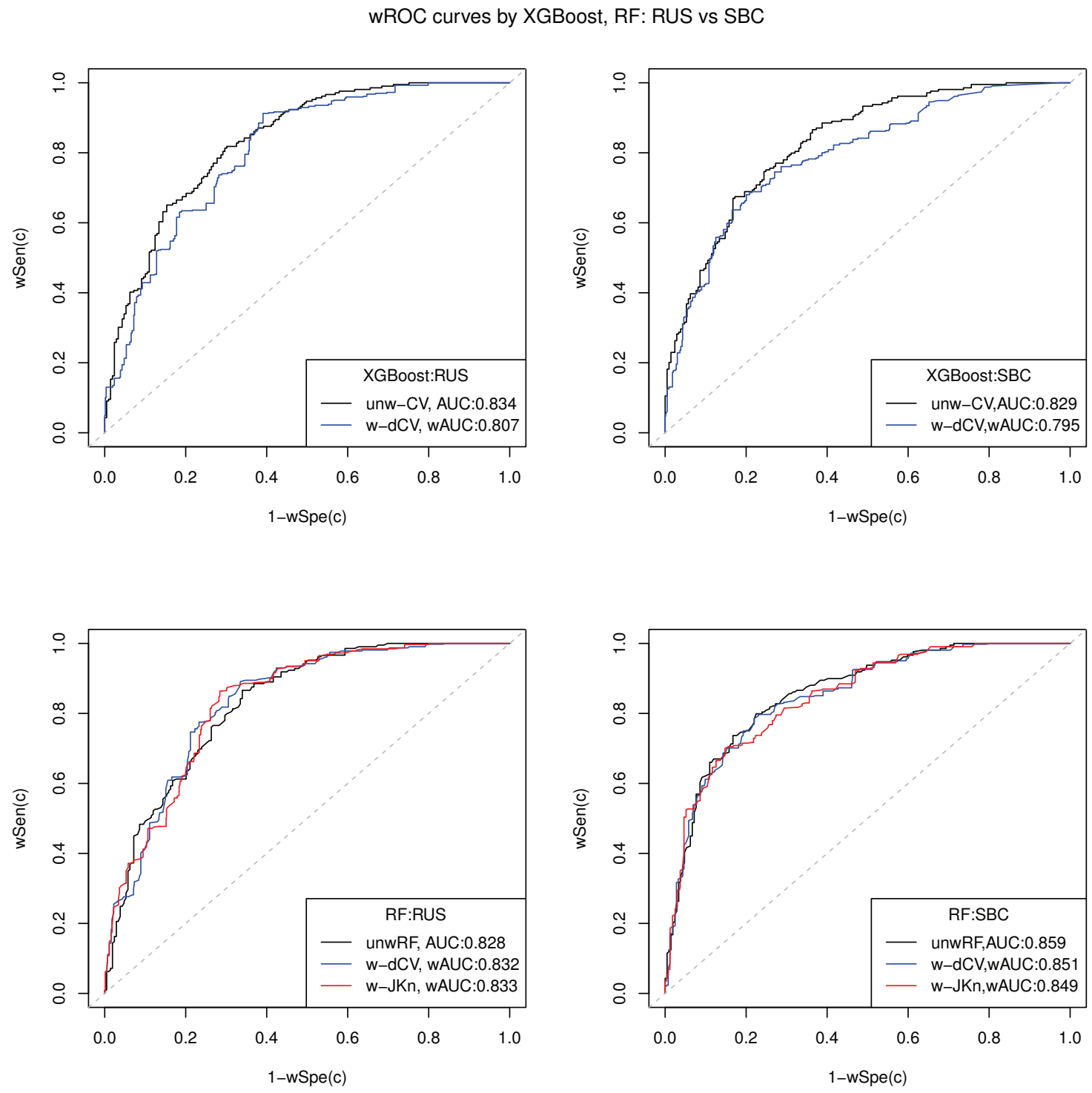

Figure S2. Diabetes: ROC and wROC curves (RUS vs SBC) for XGBoost, RF. wROC curves for unweighted models labelled as 'unw-CV' are ROC curves, i.e. AUC=wAUC.

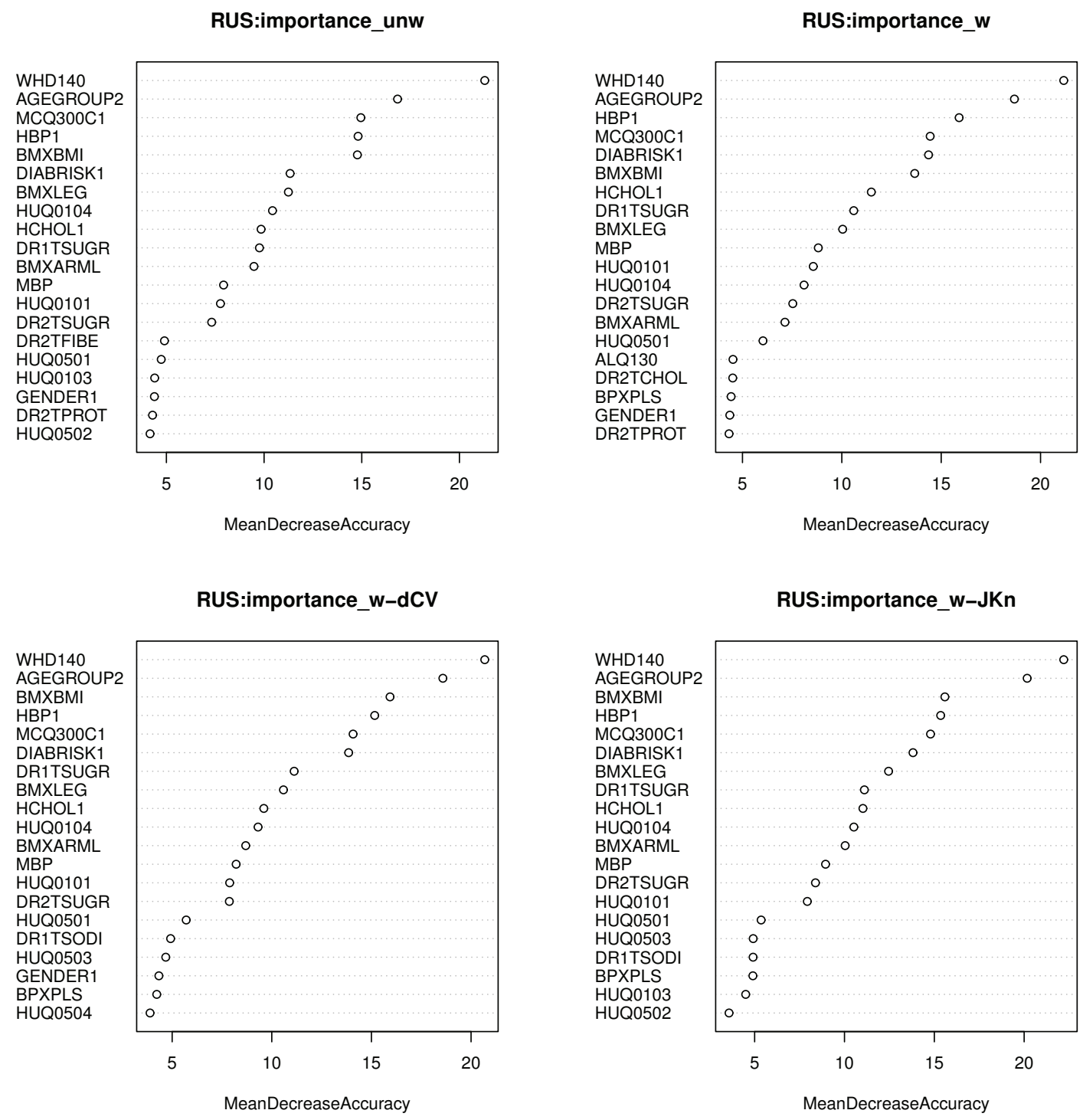

Figure S3. Diabetes: Variable selection (RUS) by RF. 'importance_unw' is modelled by unwRF and 'importance_w' is modelled by wRF with the strata option.

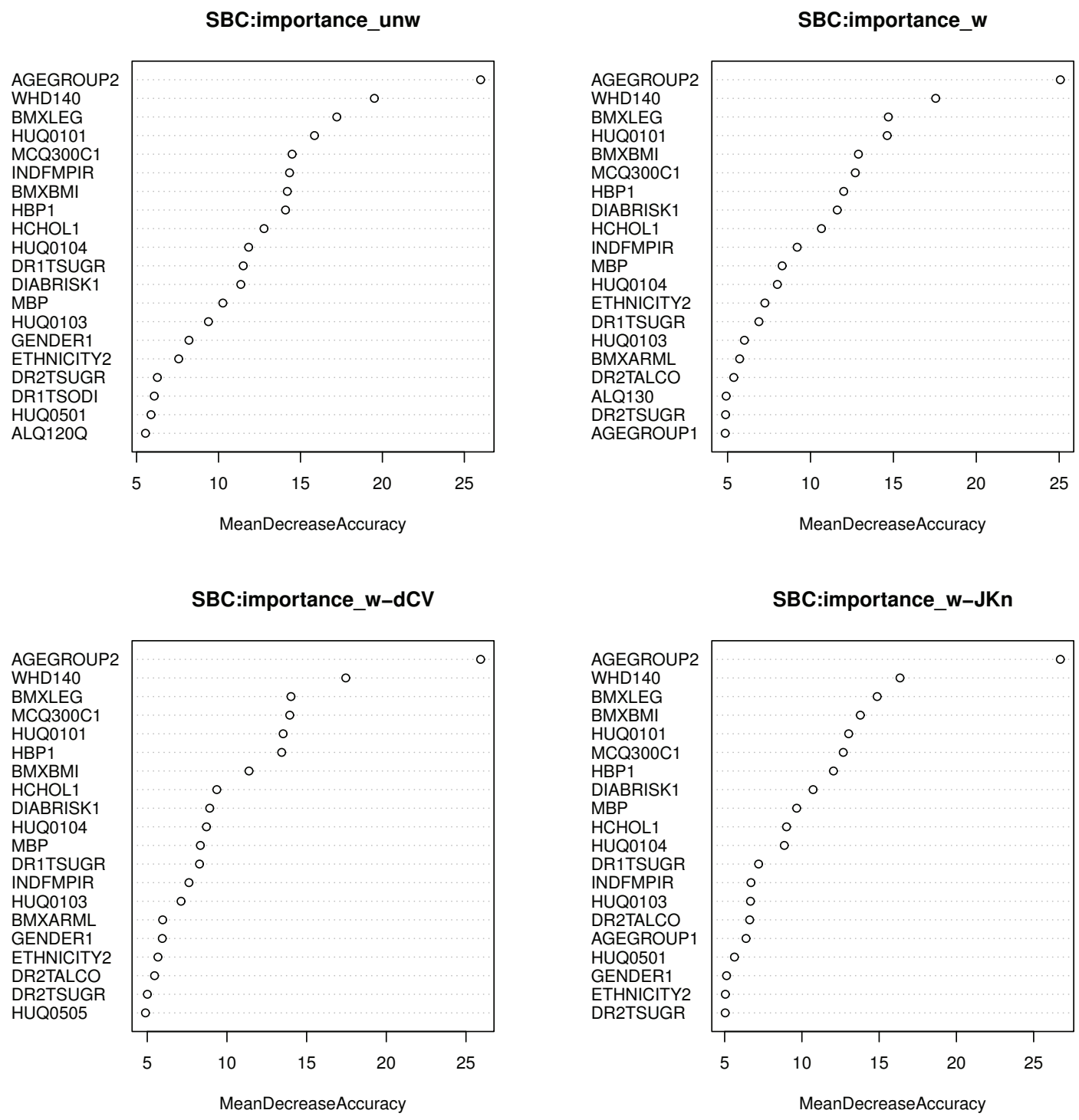

Figure S4. Diabetes: Variable selection (SBC) by RF. ‘importance_unw’ is modelled by unwRF and ‘importance_w’ is modelled by wRF with the strata option.

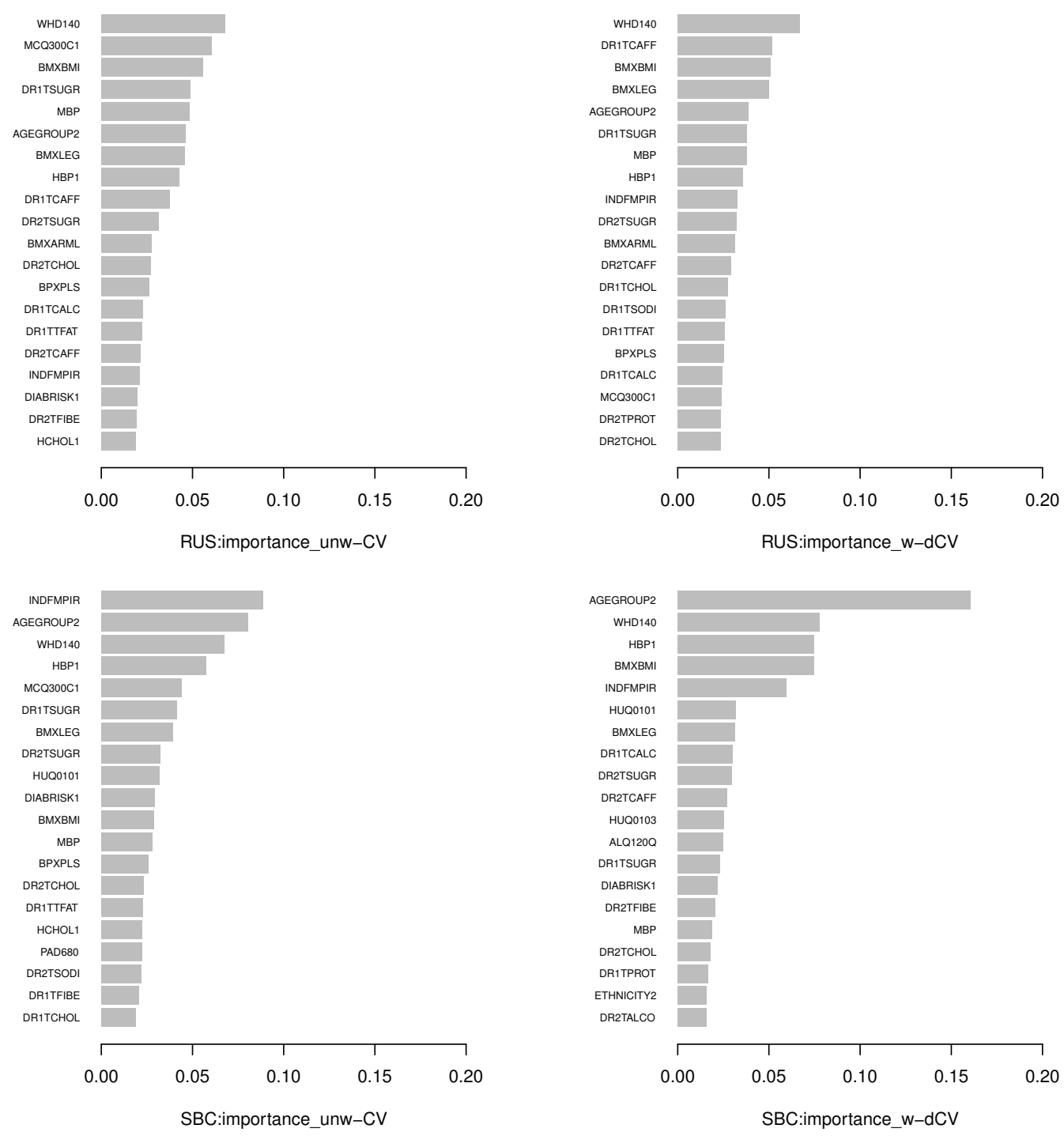

Figure S5. Diabetes: Variable selection (RUS vs SBC) by XGBoost.
